# Supplementary material for: Are carbon emissions trading and green financial instruments synergistic? -Comprehensive quantitative research based on content analysis
Source: PLoS One. 2024 Mar 7;19(3):e0298601. doi: 10.1371/journal.pone.0298601 (PMC10919601; doi:10.1371/journal.pone.0298601)
Supplement: S1 Data — (ZIP) [file pone.0298601.s001.zip › Data/地级市Z_F工作报告.zip!/║╙─╧/Σ≡║╙╩╨/└┤╫╘╣1⁄2╓┌║┼í╛┬φ┐╦╩2╛▌═°í┐_2008.docx]

工作回顾

--综合实力迈上新台阶。初步核算,全市生产总值达到446.6亿元,比2006年增长13.8%;人均生产总值突破2400美元,高于全省平均水平200美元;规模以上工业增加值220.9亿元,增长21.4%;城镇以上固定资产投资159.7亿元,增长38.8%;粮食总产量达到156.1万吨,成为历史上第二个丰收年;地方财政一般预算收入18.2亿元,增长29.5%;社会消费品零售总额127.9亿元,增长16.7%。

--城市面貌发生新变化。沙澧河开发建设开局良好,"五城同创"全面启动,新区开发和旧城改造力度加大,城市基础设施不断完善,绿化、亮化、美化水平得到提升。

--县区发展呈现新活力。各县区抓招商、上项目、兴产业,发展欲望强烈,工作局面活跃。县区生产总值占全市经济总量的比重达到80.1%;县区税收增长36%,较上年增长5.5个百分点。舞阳、临颍地方财政收入首次突破2亿元。

--居民收入实现新突破。城镇居民人均可支配收入10732元、增长18.8%,农村居民人均纯收入4567元、增长17.6%,城乡居民储蓄存款余额170.4亿元,群众生活明显改善。

--漯河形象得到新提升。成功举办了第二届中博会食品分会暨第五届中原食品节、河南省第二届豫菜品牌大赛,全国畜牧业生产形势分析会、农村人口文化现场会等十多个国家级、省级现场会相继在我市召开。我市成为全国首家农业综合标准化示范市,荣获中国特色魅力城市和中部最佳投资城市称号。

--社会事业取得新进步。投入11亿元用于教育、文化、卫生等事业的发展,积极解决事关人民群众切身利益的问题,一些工作走在全省、全国前列。

一年来,我们认真落实省定10件实事,切实抓好市定16件实事,各级政府抓发展的力度越来越大,重视民生问题的程度越来越高,全市上下团结拼搏,社会大局和谐稳定,各项工作都取得了新的成绩。

折叠(一)

工业发展势头强劲,特色产业优势凸显。全市新增规模以上工业企业82家,总数达511家。全部工业实现增加值280.2亿元,占全市生产总值的62.7%,工业对全市经济的贡献率达81.1%。"重点工业超千亿计划"进展顺利。81家重点企业实现销售收入721.2亿元、利润72亿元,分别占规模以上工业的76.2%和66.9%。主导产业快速发展。中国食品名城的品牌效应不断发挥,规模以上食品工业增加值占规模以上工业增加值的52.2%,实现销售收入626.2亿元、利税89.4亿元,分别增长33.6%、42.3%。双汇集团销售收入突破300亿元。造纸工业实现销售收入62.9亿元、利税9.7亿元,分别增长10.7%、10.4%。盐化工业实现销售收入14.9亿元、利税3.8亿元,分别增长80.1%、116.7%。中小企业发展呈现新活力。完善中小企业发展政策,组建正信、金鼎担保公司,积极打造投融资平台,推动中小企业快速发展。全市中小企业完成工业增加值138.5亿元,占全市工业的"半壁江山"。科技工作和实施名牌战略取得新成果。9个科技攻关项目得到省以上支持,科技进步对经济增长的贡献率达47.2%。新培育省级企业技术中心2家、高新技术企业6家、高新技术产品8个,有4项成果获省科技进步奖。拥有中国名牌产品4个、中国免检产品8个、河南省名牌产品24个,名优产品数量位居全省前列。

折叠(二)

"三农"工作全面加强,新农村建设富有成效。支农惠农政策全面落实。共发放粮食直补、价格综合直补、良种补贴、农机购置补贴等各类资金4.9亿元。农业结构进一步优化。优质粮面积占78.4%,畜牧业增加值占农业增加值的46.2%,粮经比例达到66:34,新增省以上农业产业化龙头企业9家,龙头企业数量、无公害农产品产地认定和产品认证数量均居全省前列。强力推进"双汇产业化工程",新建畜牧养殖场、养殖小区102个,经国家批准备案出口的养殖场589家,占全省的80%、全国的32%。全年生猪出栏403万头,增速居全省第1位。完成造林面积6.8万亩,林木覆盖率达到20.1%。农村生产生活条件得到改善。"两县十镇百村示范带动工程"顺利推进,新建户用沼气池4万多座,新解决11.6万人饮水安全问题,新建改造农村公路377公里、农家店248家,小麦机收面积占播种面积的98%,玉米秸秆机械还田面积占播种面积的60%以上,新建50个综合信息服务示范村。农田水利建设、农业综合开发、农作物秸秆禁烧及综合利用工作取得明显成效。

折叠(三)

改革开放不断深化,发展活力显著增强。企业改革成效明显,双汇集团等几家重点企业全面完成战略重组。非公有制经济快速发展,完成增加值占全市生产总值的56%。财税、金融、供销、粮食流通等改革取得实效。积极构建投融资平台,城信社申办商业银行进展顺利。高效行政服务机制建设深入推进。行政事业单位国有资产管理得到加强。对外开放迈出新步伐。第二届中博会食品分会暨第五届中原食品节签约项目达103个,总投资163亿元,已落地项目90个,履约率达到87.4%。全年新批外商投资项目17个,实际利用外资1.7亿美元、增长79.5%,实际利用省外资金41.4亿元、增长61.7%。外贸出口9061万美元,一批境内外知名企业在我市落户。项目建设力度加大,市定70个重点项目完成投资73.6亿元。

折叠(四)

生态宜居名城建设扎实推进,城镇化和城乡一体化水平不断提高。沙澧河开发建设取得阶段性成效。坚持国际标准,面向全球招标,全面完成了规划设计方案。一期拆迁顺利完成,安置房建设接近尾声,澧河一期工程快速推进。和谐拆迁的做法,受到省委、省政府充分肯定,徐光春书记作出长篇批示,多家新闻媒体进行大剂量报道。城市基础设施和环境质量明显改善。新建、改造市区主干道路和背街小巷33条,新建垃圾中转楼5座,改造水冲式厕所45座,一批城市供水、供气、供热和环卫基础设施得到完善。市区绿化覆盖率达到40.3%,高于全省平均水平。城市空气质量优良天数达340天,城市环境综合整治定量考核位居全省第二。城乡一体化发展步伐加快。市区建成区面积达到51平方公里,市区常住人口达到50万人。临颍、舞阳县城规模不断扩大,小城镇建设步伐加快,全市城镇化率达到35.7%,提高2.4个百分点。

折叠(五)

社会建设力度加大,各项事业全面进步。教育事业协调发展。全市共发放农村义务教育"两免一补"资金8376万元,投入危房改造资金1663万元、课桌凳更新配置资金1584万元,"两基"工作通过国家验收。漯河高中新校区建成招生,漯河医专顺利通过教育部评估,漯河职院被评为全省示范性高职院校。文化事业健康发展。科教文化艺术中心、新闻大厦建成投用,漯河心意六合拳、舞阳农民画、临颍双人旱船舞入选全省第一批非物质文化遗产。乡镇文化站覆盖率达100%,村文化大院达到50%以上。城乡医疗卫生服务体系不断完善。在全省率先实现了新型农村合作医疗全覆盖,参合率达95.5%;乡镇卫生院扩建项目全部完工,新建标准化村卫生室512个。我市连续三年荣获全国无偿献血先进市称号。体育事业蓬勃开展。我市成为国家青少年排球训练基地,承办国际国内大型赛事12项。全民健身运动健康发展。社会保障能力明显增强。新增城镇就业5.39万人,落实再就业资金5100万元,发放小额担保贷款3500万元。免费培训下岗失业人员9925人,下岗失业人员再就业2.1万人。城镇登记失业率控制在2.09%,低于省控目标2.4个百分点。新开工建设经济适用房21万平方米,向1500多名职工发放个人住房公积金贷款1.5亿元。农村五保户集中供养8110人,集中供养率60%,居全省第一。

切实保障妇女、未成年人、老年人和残疾人的合法权益,关爱女孩和农村留守儿童工作走在全国前列,信息化建设全国先进,食品药品监管工作全省优秀,计划生育、环境保护和土地管理三项基本国策得到较好落实。节能减排取得新成效,在全省率先全部拆除粘土砖瓦窑场,土地复垦工作扎实推进。人防工程建设创出新模式,统计工作数据质量得到提高,气象工作预测预报能力不断增强,广播电视外宣工作成效明显,旅游开发势头良好,史志档案、民族宗教、外事侨务、新闻出版等都取得新成绩。

折叠(六)

民主法制建设不断加强,社会局势持续稳定。严格执行人大及其常委会的各项决议、决定,自觉接受人大的工作监督、法律监督和政协的民主监督,610件人大代表议案、建议和政协委员提案全部办结,按时办结率达100%。审计工作力度加大,廉政建设得到加强。法治城市建设成效明显,荣获全省平安建设先进市、安全生产优秀市。应急管理体系初步形成。社会治安防范能力综合评价和公共安全感指数全省第一。稳定工作全省领先,是全省唯一无赴京到省非正常上访"双零市"。民兵预备役、双拥工作和国防教育扎实推进,驻漯解放军、武警和民兵预备役人员在维护社会稳定、支持地方经济建设中做出了重要贡献。

各位代表,以上成绩的取得,是省委、省政府和市委正确领导的结果,是人大、政协大力支持的结果,是全市人民抢抓机遇、共同奋斗的结果。在此,我代表市人民政府,向全市广大工人、农民、知识分子、各级干部和离退休老同志,向驻漯解放军指战员、武警官兵、公安干警和中央、省属驻漯单位,向各民主党派、工商联、人民团体和各界人士,向所有关心支持漯河发展的同志们、朋友们,致以崇高的敬意和衷心的感谢!

各位代表,回顾一年来的工作,我们深深体会到:只有坚持科学发展观,解放思想,大胆探索,才能实现经济社会又好又快发展;只有坚持改革创新,放眼世界,开放兼容,才能使我们的事业充满生机和活力;只有坚持依法行政,强化监督,阳光运作,才能保证人民赋予的权力始终用来为人民谋利益;只有坚持以民为本,依靠人民,服务人民,才能得到人民群众的拥护和支持!

我们也清醒地认识到,面临的严峻形势不容忽视,经济社会发展中存在的突出矛盾和问题依然较多。经济总量还比较小,增速还不够快;轻重工业结构不均衡,重工业占比较低,第三产业发展滞后;新兴产业和高新技术产业发展不快,企业自主创新能力不强;经济的外向度偏低,外贸出口增速较慢;县域经济实力不强;部分低收入群众生活还很困难,一些社会保障等问题群众还不很满意,一些影响社会稳定的因素依然存在;优化环境任务依然繁重,少数部门服务意识不强,办事效率不高,转变政府职能的任务依然艰巨;因遭受严重自然灾害,部分计划指标完成得不够理想。对此,我们一定要始终保持清醒头脑,以对漯河发展和全市人民高度负责的使命感和紧迫感,采取更加有力措施,切实解决这些问题,决不辜负人民的期望!

折叠编辑本段工作安排

今年,政府工作的总体要求是:以党的十七大精神为指导,深入贯彻落实科学发展观,坚持工业强市,坚持外向带动,加快推进工业化、城镇化和农业现代化,切实加强生态环境保护,更加注重改善民生,又好又快推进"两个率先",努力实现思想大解放、经济大发展、城市大变样、社会更和谐。

今年的主要目标是:生产总值增长13%;地方财政一般预算收入增长15%;全社会固定资产投资增长18%;社会消费品零售总额增长15%;实际利用外资增长25%,进出口总额增长16%;城镇居民人均可支配收入增长8%,农民人均纯收入增长8%;单位生产总值能耗、化学需氧量和二氧化硫排放量、人口自然增长率均控制在省定目标以内,城镇登记失业率控制在4%以内。

围绕上述目标,今年要着力抓好六个方面的重点工作:

(一)牢固坚持工业强市,加快推进工业化进程。坚持不懈地推动重点企业上台阶,中小企业上规模,抓项目、抓投入、抓技改,加快工业经济量的扩张和质的提升。一是着力壮大支柱产业和龙头企业。做大做强食品工业,快速发展造纸工业,着力培育盐化工业。深入推进"重点工业超千亿"计划,86家重点工业企业销售收入突破800亿元。强力实施"双百工程",确保100户工业企业完成生产性固定资产投资100亿元以上,特别要着力抓好2×30万千瓦热电联供、双汇5000万只肉鸡加工、永银盐化工、颍青煤化工等重大项目。力争一批重组项目取得实质性进展。二是着力振兴传统产业和中小企业。制定促进传统产业发展的政策措施,通过嫁接改造,盘活存量资产,使制革制鞋、纺织、机械制造等传统产业实现"二次振兴"。要创造条件使一些发展缓慢的老企业变"新",使一些困境企业变"活"。健全完善中小企业服务体系,注重发挥好投资担保公司的作用,建成一批中小企业创业基地,形成创业园区。重点帮扶一批高成长型中小企业,促其快速壮大。加大资本市场运作力度,抓好上市后备企业上市准备工作。三是着力培育高新技术企业。以生物医药、新材料等有一定基础和比较优势的行业为重点,培育省级以上高新技术企业3家、名牌产品和优质产品10个。四是着力抓好工业园区建设。加快市食品工业园、舞阳盐化工业园、临颍煤化工业园等园区建设,大力推行标准厂房,加快产业集聚发展、集约发展、配套发展,加快形成一批辐射带动能力强、特色突出的产业集群和产业基地。

(二)扎实做好"三农"工作,加快推进新农村建设。坚持把解决好"三农"问题作为重中之重,促进农业稳定发展、农民持续增收。大力发展现代农业。深入实施"双汇产业化工程",完善龙头企业与农户之间利益联结机制,倾斜财力重点支持规模养殖场和养殖小区。切实做好能繁母猪、奶牛补贴保险和重大动物疫情防控资金的落实,加快建设在全国有影响的畜牧业大市、畜牧业强市。全面实施农业标准化生产,大力发展特色种植、特色养殖,建设优质粮食、绿色蔬菜、苗木花卉等产业区。积极发展生态农业、城郊农业和观光旅游农业,培育出一批典型村。加快林业生态市建设,完成植树造林11.4万亩。继续抓好秸秆禁烧和综合利用。积极扶持农民专业合作组织,引导各类市场主体参与农业产业化经营。切实加强农业基础设施建设。推广农业节水设施和技术,抓好农业综合开发。加大土地复垦整理力度,加快沃土工程实施步伐。做好先进实用、生产急需农业机械的推广应用。努力改善村容村貌。全面落实省定三年规划,扎实推进"两县十镇百村示范带动工程",增加并整合支农资金,新修、改造农村公路260公里,新解决6万人饮水安全问题,新建户用沼气池4万座、农家店150家,改善农民生产生活条件,加快新农村建设步伐。多方拓宽农民增收渠道。全面落实强农惠农政策,及时足额兑现补贴资金。积极发展劳务经济,抓好农民培训,引导农民向二、三产业转移。切实加大农业投入,今年财政支农投入的增量要明显高于上年,固定资产投资用于农村的增量要明显高于上年,政府土地出让收入用于农村建设的增量要明显高于上年,千方百计让广大农民得到更多的实惠!

(三)大力调整产业结构,加快推进第三产业发展。大力发展商贸业。培育繁荣市区核心商业圈,大力发展专业批发市场,支持县、乡、村培育特色专业市场、农贸市场和农村超市。强力发展现代物流业。制定物流业发展规划,支持双汇物流等企业快速发展,加快物流口岸建设,构建漯河物流体系,带动第三产业发展。着力发展其他服务业。积极发展金融保险业。规划建设"中华名吃一条街",提高中国食品名城知名度。整合旅游资源,大力发展商务休闲业,繁荣文化市场,发展以滨河风情、城市景观、美食娱乐、演艺文化为主要内容的休闲度假旅游。加强与国内外知名会展企业的合作,积极承接区域性、专业性会展项目,促进会展业发展。以基础网络建设和信息资源开发利用为核心,着力发展信息服务业。鼓励文化传媒、信息咨询、广告设计等创意产业发展,努力使商贸业繁荣起来,物流业壮大起来,整个第三产业都振兴起来!

(四)大胆创新发展理念,加快推进改革开放。漯河改革开放的实践证明,发展上的每一个重大突破,工作上的每一个重大进步,都是解放思想、改革创新的结果。推动科学发展,最大障碍是思想不够解放,最大束缚是观念落后于实践,漯河能不能增创发展新优势,又好又快全面推进更高水平的发展,关键在于思想解放能不能先人一步、改革创新能不能快人一拍。所以,我们必须更加坚定地解放思想、深化改革、扩大开放,做到不为过去的成绩而自满,不为既有的经验所束缚,不为传统的模式所局限,在创新发展上迈出更加坚定的步伐。一是思想观念再创新。思想解放的关键在干部,各级干部特别是领导干部要带头拓宽视野,跳出漯河看漯河,善于谋划发展、创新举措、抢抓机遇,一定再鼓勇气,置身开放前沿,胆子再大一点,步子再快一点,开放程度再高一点。二是改革力度再加大。深化财税改革,优化支出结构。加快市商业银行组建步伐。推进城市公用事业改革,鼓励民营资本进入城市公用事业领域。深化行政管理体制和社区管理体制改革。加快文化事业、文化体制改革步伐。积极探索农村土地流转新路子,深入推进集体林权制度改革。三是招商机制再完善。大力实施产业招商、以商招商、项目代理招商,依托名人名企名牌招商,推进职业化招商。建立政府引导,以专业队伍为骨干、以企业为主体、以中介组织为辅助的招商引资工作机制。高标准办好2008中国(漯河)食品博览会、"中国食品名城(漯河)香港行"。四是引资质量再提高。坚持"招商引资"与"择商选资"并重,更加注重产业实际和资源保护,节约集约用地,提高投资强度,杜绝高成本、高能耗、高污染、低效益项目,着力引进科技含量高、产业带动力强的项目。五是出口创汇再突破。大力优化出口产品结构,培育新的出口增长点。积极发展对外劳务合作,不断拓展对外开放的广度和深度。

(五)致力打造生态宜居名城,加快推进城乡一体化。以沙澧河开发建设为龙头,通过大投入,实施大工程,形成大亮点,实现大变样,全面提升城市品位,让漯河变得更加秀丽,让全市人民更加热爱漯河,让外来的人都喜欢这座城市。今年要突出抓好"十大工程":一是沙澧河开发建设工程。按照滨水区城市规划,启动部分区段景观工程建设和沙澧河两岸成熟地段开发建设。加快推进太行山路沙河桥、澧河桥和沙河节制闸建设,完成黄河路沙河桥建设。如期完成澧河段一期工程和安置小区建设。二是道路升级改造及交通枢纽工程。高标准改造淞江路、嵩山路等市区主干道,新建滦河路、燕山北路等市区道路,改造背街小巷21条。实施京珠高速公路漯河段改扩建工程,加快市区部分客运、货运场站建设。三是街道综合改造工程。对人民路、交通路等区域建筑立面进行改造,形成品位高雅、建筑秀美、充满生机的街道景观。同步实施街道配套设施改造,全面提升街道建设品位。四是环保基础工程。实施公厕改造,按照标识化、人性化、标准化的原则,高标准新建、改造100座以上景观式公厕。建设沙北污水处理厂一期工程,将市区日处理污水规模提升到19万吨。五是城市绿化工程。深入实施"森林围城、森林进城、果树进城"计划,推进人民东路入市口绿化改造,完善淞江广场绿化系统,建设新北环路防护林带。六是城中村改造工程。坚持政府主导、市场运作、群众自愿、让利于民的原则,每个区选定1至2个城中村进行改造。七是市场建设工程。加快专业市场建设,新建市钢材市场一期工程、大型农产品批发市场。有计划地对原农贸市场进行升级改造。八是水域生态城建设工程。在市区东北部、沙河节制闸附近,建设集居住、会务、观光、游乐、美食等为一体的水域生态城。九是市容整治工程。围绕建设全省最干净、秩序最好的城市,开展市容环境综合整治,实施城市精细化管理。十是住房保障工程。加大廉租住房、经济适用房建设力度,新建经济适用房20万平方米和一批廉租住房,继续对符合条件的城市低收入家庭实施住房补贴,做到应补尽补。同时,强力推进"五城同创"活动,力争通过中国优秀旅游城市和国家卫生城市省级考核验收。

坚持规划先行,健全城市综合交通、市场发展、绿地系统等专项规划,指导各县区搞好乡村规划编制工作,构建完善的规划体系。做好建设融资工作,盘活城市资产和资源,支持民营资本参与城市建设,形成市场化运营新格局。抓好县城和重点镇建设,完善基础设施,增强服务功能,逐步使两个县城形成与市区规划衔接、功能对接、产业联接的发展格局。加大户籍改革力度,打破城乡户籍二元化结构。快速提高城镇化率,促进城乡一体化发展。
